# How Long Do We Need to Follow-Up Our Hernia Patients to Find the Real Recurrence Rate?

**DOI:** 10.3389/fsurg.2015.00024

**Published:** 2015-06-16

**Authors:** Ferdinand Köckerling, Andreas Koch, Ralph Lorenz, Christine Schug-Pass, Bernd Stechemesser, Wolfgang Reinpold

**Affiliations:** ^1^Department of Surgery, Center for Minimally Invasive Surgery, Vivantes Hospital, Academic Teaching Hospital of Charité Medical School, Berlin, Germany; ^2^Hernia Center Cottbus, Cottbus, Germany; ^3^3CHIRURGEN, Berlin, Germany; ^4^Hernia Center Cologne, PAN-Hospital, Cologne, Germany; ^5^Department of Surgery, Hernia Center, Wilhelmsburg Hospital Gross-Sand, Hamburg, Germany

**Keywords:** inguinal hernia, incisional hernia, recurrence, follow-up, reoperation

## Abstract

**Introduction:**

It is known that recurrences continue to occur after the follow-up period of 1–5 years usually used in most hernia studies. By reviewing the data in the Herniamed Hernia Registry documenting the time interval between the recurrent operation and previous inguinal hernia repair, the present study identifies the temporal course of onset of recurrence.

**Patients and Methods:**

Prospective data were recorded in the Herniamed Registry between 1 September 2009 and 4 May 2015 on a total of 145,590 patients with 171,143 inguinal hernia operations. These included 18,774 operations due to an inguinal hernia recurrence (10.94%). During the same period, prospective data were collected on 24,385 incisional hernia operations. The latter cases included 5,328 patients with a recurrent incisional hernia (21.85%).

**Results:**

Only 57.46% of all inguinal hernia recurrences occurred within 10 years of the previous inguinal hernia operation. Some of the remaining 42.54% of all recurrences occurred only much later, even after more than 50 years. The course of onset of recurrence is markedly different for incisional hernia. About 91.87% of such recurrences occur already within 10 years of the last operation.

**Conclusion:**

Ascertainment of the actual recurrence rate after hernia repair calls for a follow-up of 10 years for incisional hernia and of 50 years for inguinal hernia. The data collected can be used to give an approximate estimate with a shorter follow-up.

## Introduction

Hernia recurrence can occur immediately, early, or later in the time course following hernia repair (1). Some authors have used 5 years to separate early from late recurrence, although a specific time frame has not been firmly established (1). Early recurrence is generally related to technical (surgeon) factors (2). Late recurrences are related to hernia biology, aging, and other patient-related factors (1). Late recurrences continue to occur, but at a slightly decreased incidence (1).

Recurrence seems to develop even after a long period of time, in particular, after non-mesh repair (2). It is known that recurrence continues to occur after the follow-up period of 1–5 years usually used in most hernia studies (2). There are relatively few studies that have calculated the recurrence rates following inguinal and incisional hernia operations after a follow-up of 10 years (3–7). To date, there are no studies on recurrences after a follow-up of more than 10 years.

By reviewing the data in the Herniamed Hernia Registry documenting the time interval between the recurrent operation and the previous inguinal hernia repair, the present study identifies the temporal course of onset of recurrence and on that basis calculates the maximum follow-up time needed to track all recurrences occurring after inguinal and incisional hernia repair.

## Materials and Methods

The Herniamed Registry is a multicenter, Internet-based hernia registry (8), into which 425 participating hospitals and surgeons engaged in private practice (Herniamed Study Group) had entered data prospectively on their patients who had undergone hernia surgery.

If a patient had experienced a recurrence after inguinal or incisional hernia repair, a search was carried out in the Herniamed Registry to identify and record the type of previous operation (suture repair, mesh repair, endoscopic procedure). Besides, the time interval between the recurrent operation and previous repair was documented.

Prospective data were recorded in the Herniamed Registry between 1 September 2009 and 4 May 2015 on a total of 145,590 patients with 171,143 inguinal hernia operations. These included 18,774 operations due to an inguinal hernia recurrence (10.94%). Of these recurrences, 10,092 (53.8%) occurred after suture repair, 4,540 (24.2%) after open mesh repair, and 4,016 (21.4%) following an endoscopic procedure (unknown 0.6%). For 16,359 (87.1%) patients, this was the first recurrence, for 1,742 (9.3%) patients, the second recurrence, for 378 (2.0%) patients, the third recurrence, and for 169 (0.9%) patients, it was more than the third recurrence (unknown 0.7%).

During the same time period, prospective data were entered into the Herniamed Registry on 24,385 incisional hernia operations. Of the latter operations, 5,328 were performed for patients with a recurrent incisional hernia (21.85%). The recurrence occurred in 2,458 (46.1%) patients following suture repair, in 2,006 (37.7%) patients after open mesh repair, and in 801 (15.0%) patients following an endoscopic procedure (unknown 1.2%). For 3,945 (74.1%) patients, this was the first recurrence, for 926 (17.4%) patients, the second recurrence, for 263 (4.9%), the third recurrence, and for 135 (2.5%) patients, it was more than the third recurrence (unknown 1.1%).

## Results

Only 57.46% of all inguinal hernia recurrences occurred within 10 years of the previous inguinal hernia operation. Some of the remaining 42.54% of all recurrences occurred only much later, even after more than 50 years (Table 1). It was possible to identify 77.25% of all recurrences after 20 years of follow-up and 97.15% of all recurrences after 50 years of follow-up. The proportion of recurrences occurring each year continues to decline. While that amounted to 13.56% in the first year following inguinal hernia operation, it had dropped to 4.19–8.69% during the subsequent 2- to 5-year period, to around 4% per year during the subsequent 5- to 10-year period, to 2% during the following 10- to 20-year period, and to around 0.7% per year during the following 20- to 50-year period.

**Table 1:** **Time interval between previous and recurrent inguinal hernia repair**.

Time interval between previous and recurrent operation (years)	Patients	% of all recurrences	Total (%)
≤1	*n* = 2.539	13.56	13.56
>1–2	*n* = 1.628	8.69	22.25
>2–3	*n* = 1.028	5.49	27.74
>3–4	*n* = 785	4.19	31.93
>4–5	*n* = 1.140	6.09	38.02
>5–10	*n* = 3.641	19.44	57.46
>10–20	*n* = 3.705	19.79	77.25
>20–50	*n* = 3.726	19.90	97.15
>50	*n* = 532	2.85	100

For incisional hernia, the course of onset of recurrence is markedly different (Table 2). From the total number of all recurrences, the proportion that will have already occurred after a 10-year follow-up was 91.87%. In the case of incisional hernia, the very high proportion of 35.19% of all recurrences already occurring the first year is conspicuous. For incisional hernia, too, the proportion of recurrences from all recurrences occurring per year drops to 20.53% in the second year, to 9.98% in the third year, to 6.14% in the fourth year, and to 7.02% in the fifth year. The annual proportion observed during the subsequent 5- to 10-year period is still 2.6%, 0.59% during the following 10- to 20-year period, and only 0.07% (per year) during the subsequent 20- to 50-year period.

**Table 2:** **Time interval between previous and recurrent incisional hernia repair**.

Time interval between previous and recurrent operation (years)	Patients	% of all recurrences	Total (%)
≤1	*n* = 1.875	35.19	35.19
>1–2	*n* = 1.094	20.53	55.72
>2–3	*n* = 532	9.98	65.70
>3–4	*n* = 327	6.14	71.84
>4–5	*n* = 374	7.02	78.86
>5–10	*n* = 693	13.01	91.87
>10–20	*n* = 314	5.89	97.76
>20–50	*n* = 115	2.16	99.92
>50	*n* = 4	0.08	100

**Figure 1:**
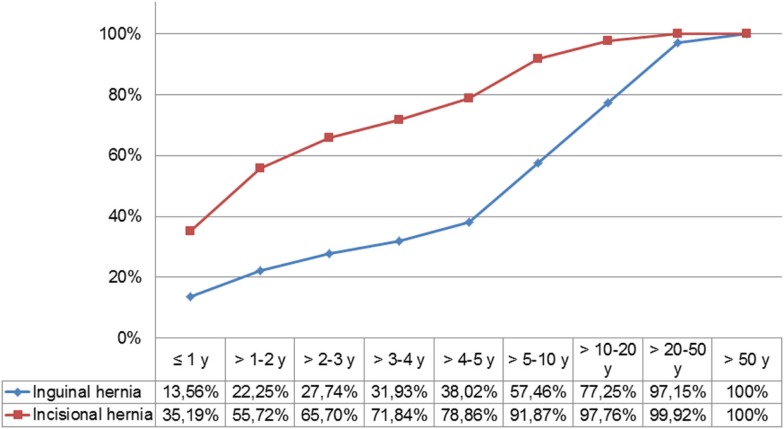
**Time line between previous and recurrent inguinal and incisional hernia repair**.

Comparison of the time lines showing the cumulative proportion of recurrences helps to identify the marked difference between inguinal hernias and incisional hernias (Figure 1). Whereas in the case of inguinal hernia, only 38.02% of recurrences had already occurred during a follow-up period of up to 5 years, 57.46% after 10 years, and 97.15% only after 50 years, for incisional hernia, already 78.86% had occurred after 5 years, and 91.83% after 10 years.

## Discussion

Based on the data presented here from the Herniamed Registry, it is possible to demonstrate that there are marked differences between inguinal hernia and incisional hernia with regard to the time interval between recurrent repair and the previous operation and that different follow-up periods are needed if one wants to calculate the actual overall recurrence rate. Whereas for incisional hernia, recurrent repair is performed much earlier and the vast majority of recurrent operations are carried out within 10 years of the previous operation, things are very different for inguinal hernia. In the latter case, only 57.46% of recurrent operations are performed within 10 years. Hence, the remaining 40% of all recurrent operations will have to be carried out after 10–50 years. This means that even with a follow-up of 10 years after inguinal hernia operation it will still not be possible to ascertain the actual recurrence rate. Onset of up to 40% of all recurrences that actually occur must still be expected. Hence, all studies with a follow-up of <10 years for incisional hernia and of <50 years for inguinal hernia only give a snapshot of the actual situation. The time lines calculated here for the proportions of recurrent operations in a large patient collective can thus serve as a basis for an approximate estimate of the mean recurrence rates in hernia studies that have a shorter follow-up. As such, recurrence rates of 1.2% with a 1-year follow-up after inguinal hernia would suggest a recurrence rate of 1.2%/13.56% × 57.46% = 5.01% after 10 years’ follow-up. For incisional hernia with a follow-up of 1 year and a documented recurrence rate of 6%, the rate would be 6%/35.19% × 91.87% = 15.7% after 10 years. On the basis of these data, realistic recurrence rates can be calculated after surgical repair of inguinal and incisional hernia. This is useful since the majority of published studies on hernia surgery only have a follow-up period of 1–5 years (2), and studies with a 10-year follow-up are an absolute exception (3–7). As a consequence of our own data, we will extend the follow-up of the patients with inguinal hernia repair in the Herniamed Registry to a minimum of 20 years.

In summary, it can be stated that the actual recurrence rates after inguinal hernia operations can only be calculated only after a 10-year follow-up, and after inguinal hernia operations only after a 50-year follow-up. Since it is hardly possible to implement such a study design in reality, the information presented here based on data from the Herniamed Registry can serve as a basis for an approximate estimate of the expected recurrence rates.

## Conflict of Interest Statement

Ferdinand Köckerling – Grants to fund the Herniamed Registry from Johnson&Johnson, Norderstedt, Karl Storz, Tutlingen, PFM Medical, Cologne, Dahlhausen, Cologne, B Braun, Tutlingen, MenkeMed, Munich and BARD, Karlsruhe. Andreas Koch, Christine Schug-Pass, Ralph Lorenz, Bernd Stechemesser, and Wolfgang Reinpold have no conflicts of interest or financial ties to disclose.

## Appendix

### Herniamed study group

#### Scientific Board

Köckerling, Ferdinand (Chairman) (Berlin); Berger, Dieter (Baden-Baden); Bittner, Reinhard (Rottenburg); Bruns, Christiane (Magdeburg); Dalicho, Stephan (Magdeburg); Fortelny, René (Wien); Jacob, Dietmar (Berlin); Koch, Andreas (Cottbus); Kraft, Barbara (Stuttgart); Kuthe, Andreas (Hannover); Lippert, Hans (Magdeburg): Lorenz, Ralph (Berlin); Mayer, Franz (Salzburg); Moesta, Kurt Thomas (Hannover); Niebuhr, Henning (Hamburg); Peiper, Christian (Hamm); Pross, Matthias (Berlin); Reinpold, Wolfgang (Hamburg); Simon, Thomas (Sinsheim); Stechemesser, Bernd (Köln); Unger, Solveig (Chemnitz).

#### Participants

Ahmetov, Azat (Saint-Petersburg); Alapatt, Terence Francis (Frankfurt/Main); Anders, Stefan (Berlin); Anderson, Jürina (Würzburg); Arndt, Anatoli (Elmshorn); Asperger, Walter (Halle); Avram, Iulian (Saarbrücken); Barkus; Jörg (Velbert); Becker, Matthias (Freital); Behrend, Matthias (Deggendorf); Beuleke, Andrea (Burgwedel); Berger, Dieter (Baden-Baden); Bittner, Reinhard (Rottenburg); Blaha, Pavel (Zwiesel); Blumberg, Claus (Lübeck); Böckmann, Ulrich (Papenburg); Böhle, Arnd Steffen (Bremen); Böttger, Thomas Carsten (Fürth); Borchert, Erika (Grevenbroich); Born, Henry (Leipzig); Brabender, Jan (Köln); Brauckmann, Markus (Rüdesheim am Rhein); Breitenbuch von, Philipp (Radebeul); Brüggemann, Armin (Kassel); Brütting, Alfred (Erlangen); Budzier, Eckhard (Meldorf); Burghardt, Jens (Rüdersdorf); Carus, Thomas (Bremen); Cejnar, Stephan-Alexander (München); Chirikov, Ruslan (Dorsten); Comman, Andreas (Bogen); Crescenti, Fabio (Verden/Aller); Dapunt, Emanuela (Bruneck); Decker, Georg (Berlin); Demmel, Michael (Arnsberg); Descloux, Alexandre (Baden); Deusch, Klaus-Peter (Wiesbaden); Dick, Marcus (Neumünster); Dieterich, Klaus (Ditzingen); Dietz, Harald (Landshut); Dittmann, Michael (Northeim); Dornbusch, Jan (Herzberg/Elster); Drummer, Bernhard (Forchheim); Eckermann, Oliver (Luckenwalde); Eckhoff, Jörn/Hamburg); Elger, Karlheinz (Germersheim); Engelhardt, Thomas (Erfurt); Erichsen, Axel (Friedrichshafen); Eucker, Dietmar (Bruderholz); Fackeldey, Volker (Kitzingen); Farke, Stefan (Delmenhorst); Faust, Hendrik (Emden); Federmann, Georg (Seehausen); Feichter, Albert (Wien); Fiedler, Michael (Eisenberg); Fischer, Ines (Wiener Neustadt); Fortelny, René H. (Wien); Franczak, Andreas (Wien); Franke, Claus (Düsseldorf); Frankenberg von, Moritz (Salem); Frehner, Wolfgang (Ottobeuren); Friedhoff, Klaus (Andernach); Friedrich, Jürgen (Essen); Frings, Wolfram (Bonn); Fritsche, Ralf (Darmstadt); Frommhold, Klaus (Coesfeld); Frunder, Albrecht (Tübingen); Fuhrer, Günther (Reutlingen); Gassler, Harald (Villach); Gawad, Karim A. Frankfurt/Main); Gerdes, Martin (Ostercappeln); Gilg, Kai-Uwe (Hartmannsdorf); Glaubitz, Martin (Neumünster); Glutig, Holger (Meissen); Gmeiner, Dietmar (Bad Dürrnberg); Göring, Herbert (München); Grebe, Werner (Rheda-Wiedenbrück); Grothe, Dirk (Melle); Gürtler, Thomas (Zürich); Hache, Helmer (Löbau); Hämmerle, Alexander (Bad Pyrmont); Haffner, Eugen (Hamm); Hain, Hans-Jürgen (Gross-Umstadt); Hammans, Sebastian (Lingen); Hampe, Carsten (Garbsen); Harrer, Petra (Starnberg); Heinzmann, Bernd (Magdeburg); Heise, Joachim Wilfried (Stolberg); Heitland, Tim (München); Helbling, Christian (Rapperswil); Hempen, Hans-Günther (Cloppenburg); Henneking, Klaus-Wilhelm (Bayreuth); Hennes, Norbert (Duisburg); Hermes, Wolfgang (Weyhe); Herrgesell, Holger (Berlin); Herzing, Holger Höchstadt); Hessler, Christian (Bingen); Hildebrand, Christiaan (Langenfeld); Höferlin, Andreas (Mainz); Hoffmann, Henry (Basel); Hoffmann, Michael (Kassel); Hofmann, Eva M. (Frankfurt/Main); Hopfer, Frank (Eggenfelden); Hornung, Frederic (Wolfratshausen); Hügel, Omar (Hannover); Hüttemann, Martin (Oberhausen); Huhn, Ulla (Berlin); Hunkeler, Rolf (Zürich); Imdahl, Andreas (Heidenheim); Jacob, Dietmar (Berlin); Jenert, Burghard (Lichtenstein); Jugenheimer, Michael (Herrenberg); Junger, Marc (München); Käs, Stephan (Weiden); Kahraman, Orhan (Hamburg); Kaiser, Christian (Westerstede); Kaiser, Stefan (Kleinmachnow); Kapischke, Matthias (Hamburg); Karch, Matthias (Eichstätt); Keck, Heinrich (Wolfenbüttel); Keller, Hans W. (Bonn); Kienzle, Ulrich (Karlsruhe); Kipfmüller, Brigitte (Köthen); Kirsch, Ulrike (Oranienburg); Klammer, Frank (Ahlen); Klatt, Richard (Hagen); Kleemann, Nils (Perleberg); Klein, Karl-Hermann (Burbach); Kleist, Sven (Berlin); Klobusicky, Pavol (Bad Kissingen); Kneifel, Thomas (Datteln); Knoop, Michael (Frankfurt/Oder); Knotter, Bianca (Mannheim); Koch, Andreas (Cottbus); Köckerling, Ferdinand (Berlin); Köhler, Gernot (Linz); König, Oliver (Buchholz); Kornblum, Hans (Tübingen); Krämer, Dirk (Bad Zwischenahn); Kraft, Barbara (Stuttgart); Kreissl, Peter (Ebersberg); Krones, Carsten Johannes (Aachen); Kruse, Christinan (Aschaffenburg); Kube, Rainer (Cottbus); Kühlberg, Thomas (Berlin); Kuhn, Roger (Gifhorn); Kusch, Eduard (Gütersloh); Kuthe, Andreas (Hannover); Ladberg, Ralf (Bremen); Ladra, Jürgen (Düren); Lahr-Eigen, Rolf (Potsdam); Lainka, Martin (Wattenscheid); Lammers, Bernhard J. (Neuss); Lancee, Steffen (Alsfeld); Lange, Claas (Berlin); Laps, Rainer (Ehringshausen); Larusson, Hannes Jon (Pinneberg); Lauschke, Holger (Duisburg); Leher, Markus (Schärding); Leidl, Stefan (Waidhofen/Ybbs); Lenz, Stefan (Berlin); Lesch, Alexander (Kamp-Lintfort); Liedke, Marc Olaf (Heide); Lienert, Mark (Duisburg); Limberger, Andreas (Schrobenhausen); Limmer, Stefan (Würzburg); Locher, Martin (Kiel); Loghmanieh, Siawasch (Viersen); Lorenz, Ralph (Berlin); Mallmann, Bernhard (Krefeld); Manger, Regina (Schwabmünchen); Maurer, Stephan (Münster); Mayer, Franz (Salzburg); Mellert, Joachim (Höxter); Menzel, Ingo (Weimar); Meurer, Kirsten (Bochum); Meyer, Moritz (Ahaus); Mirow, Lutz (Kirchberg); Mittenzwey, Hans-Joachim (Berlin); Mörder-Köttgen, Anja (Freiburg); Moesta, Kurt Thomas (Hannover); Moldenhauer, Ingolf (Braunschweig); Morkramer, Rolf (Xanten); Mosa, Tawfik (Merseburg); Müller, Hannes (Schlanders); Münzberg, Gregor (Berlin); Mussack, Thomas (St. Gallen); Neumann, Jürgen (Haan); Neumeuer, Kai (Paderborn); Niebuhr, Henning (Hamburg); Nölling, Anke (Burbach); Nostitz, Friedrich Zoltán (Mühlhausen); Obermaier, Straubing); Öz-Schmidt, Meryem (Hanau); Oldorf, Peter (Usingen); Olivieri, Manuel (Pforzheim); Pawelzik, Marek (Hamburg); Peiper, Christian (Hamm); Peitgen, Klaus (Bottrop); Pertl, Alexander (Spittal/Drau); Philipp, Mark (Rostock); Pickart, Lutz (Bad Langensalza); Pizzera, Christian (Graz); Pöllath, Martin (Sulzbach-Rosenberg); Possin, Ulrich (Laatzen); Prenzel, Klaus (Bad Neuenahr-Ahrweiler); Pröve, Florian (Goslar); Pronnet, Thomas (Fürstenfeldbruck); Pross, Matthias (Berlin); Puff, Johannes (Dinkelsbühl); Rabl, Anton (Passau); Rapp, Martin (Neunkirchen); Reck, Thomas (Püttlingen); Reinpold, Wolfgang (Hamburg); Reuter, Christoph (Quakenbrück); Richter, Jörg (Winnenden); Riemann, Kerstin (Alzenau-Wasserlos); Rodehorst, Anette (Otterndorf); Roehr, Thomas (Rödental); Roncossek, Bremerhaven); Roth Hartmut (Nürnberg); Sardoschau, Nihad (Saarbrücken); Sauer, Gottfried (Rüsselsheim); Sauer, Jörg (Arnsberg); Seekamp, Axel (Freiburg); Seelig, Matthias (Bad Soden); Seidel, Hanka (Eschweiler); Seiler, Christoph Michael (Warendorf); Seltmann, Cornelia (Hachenburg); Senkal, Metin (Witten); Shamiyeh, Andreas (Linz); Shang, Edward (München); Siemssen, Björn (Berlin); Sievers, Dörte (Hamburg); Silbernik, Daniel (Bonn); Simon, Thomas (Sinsheim); Sinn, Daniel (Olpe); Sinning, Frank (Nürnberg); Smaxwil, Constatin Aurel (Stuttgart); Schabel, Volker (Kirchheim/Teck); Schadd, Peter (Euskirchen); Schassen von, Christian (Hamburg); Schattenhofer, Thomas (Vilshofen); Scheidbach, Hubert (Neustadt/Saale); Schelp, Lothar (Wuppertal); Scherf, Alexander (Pforzheim); Scheyer, Mathias (Bludenz); Schimmelpenning, Hendrik (Neustadt in Holstein); Schinkel, Svenja (Kempten); Schmid, Michael (Gera); Schmid, Thomas (Innsbruck); Schmidt, Rainer (Paderborn); Schmidt, Sven-Christian (Berlin); Schmidt, Ulf (Mechernich); Schmitz, Heiner (Jena); Schmitz, Ronald (Altenburg); Schöche, Jan (Borna); Schoenen, Detlef (Schwandorf); Schrittwieser, Rudolf/Bruck an der Mur); Schroll, Andreas (München); Schultz, Christian (Bremen-Lesum); Schultz, Harald (Landstuhl); Schulze, Frank P. Mülheim an der Ruhr); Schumacher, Franz-Josef (Oberhausen); Schwab, Robert (Koblenz); Schwandner, Thilo (Lich); Schwarz, Jochen Günter (Rottenburg); Schymatzek, Ulrich (Radevormwald); Spangenberger, Wolfgang (Bergisch-Gladbach); Sperling, Peter (Montabaur); Staade, Katja (Düsseldorf); Staib, Ludger (Esslingen); Stamm, Ingrid (Heppenheim); Stark, Wolfgang (Roth); Stechemesser, Bernd (Köln); Steinhilper, Uz (München); Stengl, Wolfgang (Nürnberg); Stern, Oliver (Hamburg); Stöltzing, Oliver (Meissen); Stolte, Thomas (Mannheim); Stopinski, Jürgen (Schwalmstadt); Stubbe, Hendrik (Güstrow/); Stülzebach, Carsten (Friedrichroda); Tepel, Jürgen (Osnabrück); Terzić, Alexander (Wildeshausen); Teske, Ulrich (Essen); Thews, Andreas (Schönebeck); Tichomirow, Alexej (Brühl); Tillenburg, Wolfgang (Marktheidenfeld); Timmermann, Wolfgang (Hagen); Tomov, Tsvetomir (Koblenz; Train, Stefan H. (Gronau); Trauzettel, Uwe (Plettenberg); Triechelt, Uwe (Langenhagen); Ulcar, Heimo (Schwarzach im Pongau); Unger, Solveig (Chemnitz); Verweel, Rainer (Hürth); Vogel, Ulrike (Berlin); Voigt, Rigo (Altenburg); Voit, Gerhard (Fürth); Volkers, Hans-Uwe (Norden); Vossough, Alexander (Neuss); Wallasch, Andreas (Menden); Wallner, Axel (Lüdinghausen); Warscher, Manfred (Lienz); Warwas, Markus (Bonn); Weber, Jörg (Köln); Weiss, Johannes (Schwetzingen); Weissenbach, Peter (Neunkirchen); Werner, Uwe (Lübbecke-Rahden); Wessel, Ina (Duisburg); Weyhe, Dirk (Oldenburg); Wieber, Isabell (Köln); Wiesmann, Aloys (Rheine); Wiesner, Ingo (Halle); Withöft, Detlef (Neutraubling); Woehe, Fritz (Sanderhausen); Wolf, Claudio (Neuwied); Yildirim, Selcuk (Berlin); Zarras, Konstantinos (Düsseldorf); Zeller, Johannes (Waldshut-Tiengen); Zhorzel, Sven (Agatharied); Zuz, Gerhard (Leipzig).
